# Cellular Competency during Development Alters Evolutionary Dynamics in an Artificial Embryogeny Model

**DOI:** 10.3390/e25010131

**Published:** 2023-01-09

**Authors:** Lakshwin Shreesha, Michael Levin

**Affiliations:** 1 UFR Fundamental and Biomedical Sciences, Université Paris Cité, 75006 Paris, France; 2 Allen Discovery Center, Tufts University, Medford, MA 02155, USA

**Keywords:** artificial life, in silico, artificial embryogeny, evolutionary computation, development, morphogenesis, basal cognition

## Abstract

Biological genotypes do not code directly for phenotypes; developmental physiology is the control layer that separates genomes from capacities ascertained by selection. A key aspect is cellular competency, since cells are not passive materials but descendants of unicellular organisms with complex context-sensitive behavioral capabilities. To probe the effects of different degrees of cellular competency on evolutionary dynamics, we used an evolutionary simulation in the context of minimal artificial embryogeny. Virtual embryos consisted of a single axis of positional information values provided by cells’ ‘structural genes’, operated upon by an evolutionary cycle in which embryos’ fitness was proportional to monotonicity of the axial gradient. Evolutionary dynamics were evaluated in two modes: hardwired development (genotype directly encodes phenotype), and a more realistic mode in which cells interact prior to evaluation by the fitness function (“regulative” development). We find that even minimal ability of cells with to improve their position in the embryo results in better performance of the evolutionary search. Crucially, we observed that increasing the behavioral competency masks the raw fitness encoded by structural genes, with selection favoring improvements to its developmental problem-solving capacities over improvements to its structural genome. This suggests the existence of a powerful ratchet mechanism: evolution progressively becomes locked in to improvements in the intelligence of its agential substrate, with reduced pressure on the structural genome. This kind of feedback loop in which evolution increasingly puts more effort into the developmental software than perfecting the hardware explains the very puzzling divergence of genome from anatomy in species like planaria. In addition, it identifies a possible driver for scaling intelligence over evolutionary time, and suggests strategies for engineering novel systems in silico and in bioengineering.

## 1. Introduction

One critical aspect of real biology which is not always taken into account in evolutionary computation and theoretical biology efforts, is that the mapping between genotype and phenotype is not direct [1,2,3,4,5,6,7,8,9,10,11,12,13]. Genes generally do not directly encode for structure and function of the organism. Instead, it has become increasingly clear that developmental physiology provides a critical layer of control that sits between genomes (on which mutation operates) and anatomy (the phenotype which is the subject of selection). During development, organisms emerge as the result of a complex set of interactions among cells, with anatomical order and functionality being the result of cellular activities. While genomes specify the cellular hardware (proteins), it is the software (cellular activity) studied by developmental biologists that is ultimately responsible for the organism’s overall structure and behavior [14,15,16,17,18].

The simple story of genomes determining anatomy is shown to be incomplete by examples such as the highly regenerative planaria [19]: due to reproduction by fissioning and regeneration, they retain mutations made in the parent body and pass them on to their offspring (somatic inheritance). As a result, planaria have an incredibly messy genome (indeed, worms are mixoploid—different numbers of chromosomes in each cell). Despite this, they have the most reliable anatomy: every fragment of a planarian regenerates a perfect worm each time. They are essentially immortal, and highly resistant to cancer [20]. How can the animal with the most chaotic genome have the most reliable, robust anatomy? Fundamental knowledge gaps in this area not only impede our understanding of basic evolutionary developmental biology but also limit our ability to make desired system-level changes to complex anatomy in the context of regenerative medicine [21,22,23].

The indirect relationship between genotype and phenotype has a number of important implications. For example, it is currently impossible to guess the anatomy of an organism by examining its genome—overall symmetry type, number and kinds of organs, size, regenerative capacity, etc.—can only be estimated if one compares a genome to that of another organism for which all of these are already known. Likewise, even when one has access to complete genomes, for example of the frog and axolotl, one cannot guess the shape of a chimeric embryo: will a “frogolotl”, consisting of 50% of each kind of cells, make legs (like an axolotl larva) or not (like a tadpole)? This is because, while much research has shed light on molecular mechanisms necessary for morphogenesis, the field still largely lacks an understanding of the key dynamics that determine form and function: large-scale anatomical decision-making by cellular collectives [19,24,25].

Importantly, the cells that make up these collectives evolved from independent unicellular organisms with extensive capabilities for sensing their environments and responding. The key role of these capabilities in morphogenesis reframes cells as an agential [26], not a passive, material. There are numerous examples: one of the most remarkable qualities of morphogenesis is its competency in reaching an adaptive anatomical outcome despite novel starting states and perturbations [24,27,28]. For example, mammalian embryos can be split into pieces, and each piece gives rise to a complete organism (monozygotic twinning). Some animals retain these regenerative capacities into adulthood—salamanders whose limbs (or eyes, jaws, tails, etc.) are amputated will re-grow exactly the missing portion and then stop when the correct structure is complete [29]. Tadpoles with scrambled faces become largely normal frogs, as the craniofacial organs move in novel paths until the correct configuration is achieved [30,31,32]. Tadpoles with eyes placed on their tails (and not in their heads) can see [33]. All of this means that mutations resulting in noise or changes in initial positions of the organs, which would have been disastrous for a hardwired architecture, will not have a strong effect on survival because the tissues will make needed reconfigurations to compensate for errors in initial state. It is clear that this rapid, built-in capacity for anatomical homeostasis and problem-solving must have implications for the evolutionary process, but this has not been extensively explored.

We previously proposed that the competency of the developmental layer results from the navigation policies of a collective intelligence of cells in anatomical morphospace—an evolutionary precursor to the intelligence of neural cells which are well known to navigate 3-dimensional and other problem spaces [24,27]. We refer to these navigation policies as *competency* of the cellular collective—the capacity to sense their environment and each other, and to communicate to effectively solve problems in morphospace during development (i.e., reach appropriate target morphology despite perturbations and changing internal and external conditions). Such collective cellular intelligence is an essential aspect of morphogenesis during development, regeneration, and cancer suppression, and is central to the genome-form-function relationship. As has been claimed for learning [22,34,35], these collective competencies could greatly smooth the evolutionary landscape and enable access to regions of the phenotype space that would otherwise have been hard or impossible to reach.

The evolutionary importance of information not encoded in the genome directly has been studied previously by evolutionary biologists and in the field of artificial life, primarily in the context of learning [24,27]. Many organisms can modify their behavior in light of experience, improving their fitness via information that was not provided by the genome. As early as 1896, Baldwin proposed that such adaptive behaviors, while not encoded by the genome, are assimilated into the genome (hardcoded) over evolutionary timescales, a phenomenon known as the Baldwin Effect [24,27]. Classic simulations of the interplay between learning and evolution demonstrated that the Baldwin Effect does indeed change the evolutionary landscape of organisms capable of learning [24,27]. This dynamic offers competing tendencies, in which learning takes pressure off the genome via a shielding effect [24,27] at first, by providing behaviors that do not need to be discovered by evolution of the genome. Subsequently, genetic assimilation takes the pressure off the need for learning, by eventually finding ways to hardcode those behaviors. This learning paradigm emphasizes two different kinds of fitness [24,27]. *Genotypic fitness* is the quality of the structural genome—what the organism would have been able to accomplish given only the static information in its genome. *Phenotypic fitness* is what selection actually ‘sees’—the performance of the organism after both hardwired and learned repertoires have had their chance to shine.

Here, we introduce cellular competency as another important contributor to phenotypic fitness. Cellular competency [25,34,35] has a number of important differences from learning. First, the ability of cells and tissues to attain and maintain setpoints in morphological spaces is independent of learning at the level of the individual. Second, learning at the level of the organism needs a mechanism (e.g., nervous system architecture) that must itself be painstakingly evolved—evolution must discover, maintain, and pay the costs of new capabilities such as brains and exploratory behaviors. In contrast, cellular competencies come “by default” because organisms consist of cellular components that already have many capabilities evolved during their ancestral lifetime as independent organisms. Thus, it is important to begin to study how cellular competencies affect evolution, to complement approaches focused on learning, evolvability mechanisms [36,37,38,39,40,41], and the material properties of morphological computation [42,43,44,45,46]. All of these factors interact in vivo, and will need to be studied separately and together.

The question we address here is: How do diverse levels of competency in the cellular collective during morphogenesis impact the rate and course of the evolutionary process? We undertook a quantitative investigation of this question using a minimal model of artificial embryogeny. Our model did not include learning or classical behavior at the individual level, but instead solved a problem in morphogenetic space. Our system simulates an animal with a single axis of positional information values (such as the anterior-posterior axis) [47,48]. Virtual embryos consist of a 1-dimensional array of integers, with their evolutionary fitness being proportional to the degree of monotonicity of those values. In the baseline case, we use a direct encoding where the phenotype is a direct consequence of the genotype—the values of the array directly specify the order of values in each embryo. Under these conditions, a genetic algorithm eventually produces structural genes in which all the values are in the correct (monotonic) order. We compare these outcomes to a more realistic case, in which the mapping is not direct: we introduce a development algorithm in which individual cells have some degree of competency to rearrange themselves based on their local environment. Cells can move to numerically more-advantageous positions before evaluation of phenotypic fitness. We accomplish this through a restricted bubble sort procedure [49], highlighting the conceptual similarity between sorting algorithms and navigation in a geometric problem space. This corresponds to embryogenesis in vivo, in which cells act before the mature animal’s fitness is ascertained in the environment. Importantly, our system does not include Lamarckian inheritance. Instead, it features a strong barrier between soma and germline: the rearrangements occur for each individual but the only thing that gets passed on to their offspring is their original pre-swap structural genome [50,51].

We varied the degree of cellular competency, and tracked the dynamics of the resulting evolution, both in terms of raw genotypic fitness and phenotypic fitness. We also explored the effects of adding a competency cost. We observed a number of interesting outcomes. First, including a developmental layer that models a range of cellular competencies improves evolutionary efficiency in proportion to the degree of cellular competency. Second, in mixed populations, competent individuals tend to eventually dominate the population. Third, when the degree of competency is itself allowed to evolve, populations settle on a specific, sub-maximal level of competency. Finally, and most critically, we observed that because competency hides genetic deficiencies from selection, pressure to improve the structural genome is released, while pressure to improve the morphogenetic competency of cells is increased. These dynamics establish a positive feedback loop in which populations advance by progressively improving cellular capacities, not just the genes dictating the actual initial structure of each embryo. This provides an explanation for the otherwise mysterious disconnect between planarian genomes and their amazing anatomical robustness, and suggests the existence of an evolutionary ratchet working to optimize intelligence in even very basal forms [52,53,54,55,56,57].

## 2. Methods

We simulate the evolution of artificial 1-dimensional embryos in silico. The following sections describe the structure of each embryo, our paradigm for modeling developmental morphogenesis towards a target adult anatomy, and the process of selection employed to study their dynamics over time.

### 2.1. Creating Populations for Evolution

A population consists of a number of embryos. Each embryo is represented as a one-dimensional array of fixed size (matching the cell count in the 1-dimensional embryo). Each cell of this array is initialized with a different integer value representing the positional value gene for the corresponding cell of the embryonic axis (see Supplement S1). In this minimal model, there is no further chromosomal structure or transcriptional change, and we simply refer to the structural genes as directly specifying the positional preference of a given cell. Each embryo undergoes a developmental cycle (described below) to become a mature “individual”. We model evolution in three kinds of populations: a “hardwired” population consisting of only hardwired embryos, a “competent” population of only competent embryos, and a “mixed” population which contains both kinds of embryos, in varying proportions. Our mixed populations have 200 embryos, the rest have 100.

### 2.2. Hardwired and Competent Embryos

**Figure 1:**
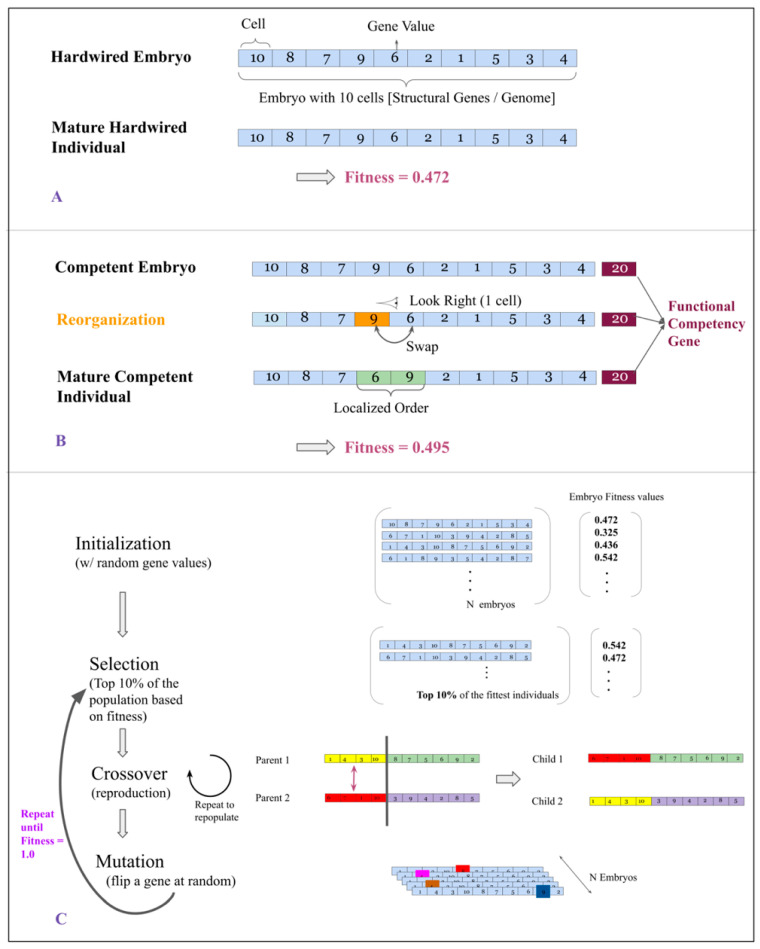
**Schematic of experimental setup**. (**A**): Definition of a hardwired embryo: Each hardwired embryo is a 1-D array consisting of 50 cells (10 shown here as example). Each cell takes an integer value between [1, 50], and is considered to be its Structural Gene. The fitness of an individual is defined as the degree of order within its genes (0 implying descending order, 0.5 implying random order and 1.0 implying ascending order). In the example shown here, the embryo is randomly initialized and hence has a fitness close to 0.5. (**B**): Definition of a competent embryo: Each competent embryo is identical to a hardwired embryo except that it carries an additional “functional” gene indicating how many cell movements it can carry out during a developmental cycle to achieve ordered ascending arrangement before phenotypic assessment. The functional gene can be locked down to a pre-specified value for an entire population or can be evolvable. (**C**): Description of the genetic algorithm used to evolve hardwired and competent embryos. See Methods for details.

We define two types of embryos, a “hardwired embryo” and a “competent embryo” (Figure 1A,B). The difference between them lies in the way they develop during the evolutionary cycle. A competent embryo consists of cells capable of sensing neighboring cells and adapting morphology by moving around prior to the adult stage in which fitness is evaluated. “Competency” is the capability of these embryos to carry out such reorganization, and they carry a gene that dictates their degree of motility (fixed, in some experiments, but free to evolve in others). Our competent embryos leverage sensing and motility to reorganize their cells during ‘development’ in a way that boosts fitness (see below and Supplement S1). We vary the degree to which they can reorganize (competency level). A hardwired embryo lacks this capability; its structure from birth to maturity is constant.

### 2.3. Developmental Cycle

Soon after initialization, embryos undergo a developmental cycle. During this process, competent (but not hardwired) embryos undergo a restricted bubble-sort procedure (see Supplement S1) to rearrange their cells in a way that boosts fitness (i.e., to increase ascending order of its array of integers). At the end of the developmental cycle, embryos are considered “individuals”.

### 2.4. Fitness of Embryos and Individuals

We define fitness as the degree to which an embryo’s array of integers is in ascending order. Individuals with cells arranged in ascending order by value are attributed a fitness of 1.0 (maximum), those whose cells are randomly ordered are attributed a fitness of 0.5. We calculate the fitness (the degree of order) of an array by counting the number of non-inversions present (see Supplement S1). At the beginning of each evolutionary cycle, all embryos are considered “just born”; their morphological structure determined by their parents from the previous generation. Therefore, we call their fitness at the start of each cycle the genotypic fitness. At the end of the developmental cycle, the fitness of each resulting individual is calculated again, which we call the phenotypic fitness. For hardwired individuals, phenotypic and genotypic fitnesses are always identical. For competent individuals, however, phenotypic fitness reflects the reorganization that occurs based on their competency level.

### 2.5. Competency Level

At the start of each developmental cycle, a competent embryo is assigned an integer representing its competency level. This integer determines how many successive bubble-sort swaps will take place during its developmental cycle. Usually, competency levels are much lower than the total number of bubble-sort swaps required by an embryo to attain maximum fitness, for this reason it is called “restricted” bubble-sort.

### 2.6. Genetic Algorithm

To evolve populations (hardwired or competent), we iteratively pass them through three stages (Figure 1C):

1. Selection: The fittest 10% of individuals in a population are selected to move on to the next generation. Selection in a population is based on its individuals’ phenotypic fitness.
2. Cross-Over: In order to repopulate a population back to its original strength, we carry out a process of reproduction called cross-over. It occurs as follows: Two individuals are involved, each of these are split at a random location along their length. One half of Individual 1 is swapped with the same half of Individual 2 to give rise to two children. Figure 1 contains an illustration of this process.
3. Mutation: The repopulated population is subjected to random point mutations. We set the probability of an individual receiving a point mutation to be 0.6.

## 3. Results

### 3.1. A Minimal System for Investigating Effects of Cellular Competency on Evolution

We built a virtual embryogeny model in which fitness was defined by the degree of monotonicity of a 1D array of numbers, simulating a minimal metazoan bodyplan—a single axis of positional information (Figure 1). The initial sequence of numbers for each embryo was assigned randomly. Since these sequences decided the embryo’s structure (cell order), they are referred to as its structural genes. As described above, in hardwired embryos, that sequence is fixed: their genome directly encoded their phenotype. For competent embryos, we implemented different degrees of competency during a developmental period in which cells were allowed some degree of movement relative to their neighbors, allowing them to reorganize to improve monotonicity prior to evaluation of phenotypic fitness. This enabled phenotypic fitness for competent individuals to diverge from raw genotypic fitness, with the extent of divergence depending on how much cell movement was permitted. This corresponds to different degrees of capacity for cells in vivo to optimize homeostatically preferred local conditions with respect to informational signals such as positional cues and polling of neighboring cell states [58]. An evolutionary cycle was implemented around these developmental events [58]. In the initial experiments, the competency gene is fixed across the evolutionary run, enabling study of the evolutionary dynamics over time as a function of different degrees of cellular competency.

### 3.2. Developmental Competency Accelerates Evolutionary Search

**Figure 2:**
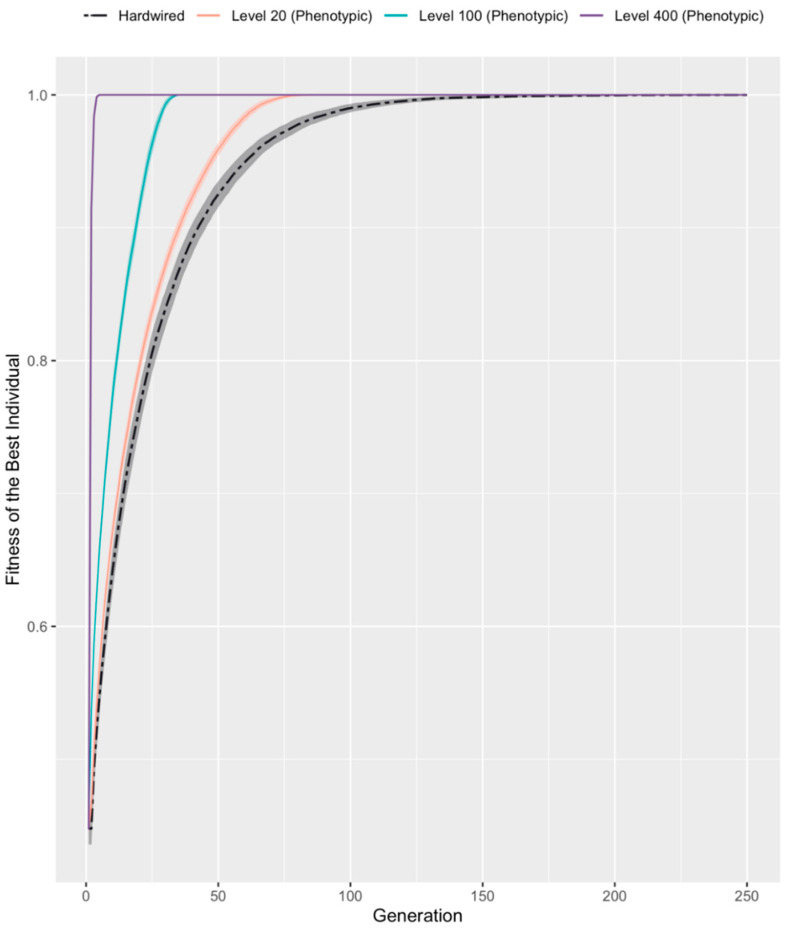
**Competent individuals have a higher rate of fitness than their Hardwired counterparts.** Three populations with different competency levels [Levels 20, 100, and 400] and a single hardwired population were initialized. Competency level refers to the maximum number of cell-swaps a competent embryo can execute during its developmental cycle. The individual with the maximum fitness in each population was plotted over 250 generations. Shaded areas represent 95% confidence interval bands over *n* = 100 repeats of each experimental condition.

We first compared, over 250 generations, the time-course of evolutionary search towards a fully ordered axis in hardwired vs. competent individuals. After 100 generations, the hardwired population had the least fitness compared to populations with varying degrees of competency (Figure 2 and Table 1). Table 1 provides a summary of the generation number at which each population crossed different fitness thresholds. We compared fitness of the best individual in competent and hardwired populations at generations 2, 10, and 20 (because these points exhibited the greatest sample variances.) At each of these, the difference in fitness between hardwired and competent populations was significant (*p*-values << 1 × 10^−3^ for all points, Student’s *t*-test; for details see Supplement S2.1.1).

Figure 2 also shows that the 95% confidence interval bands over 100 repeat runs decreased with increasing competency level, suggesting that more competent architectures are also more consistent in performance over time. Note that hardwired individuals gradually improved to reach peak fitness, taking well over 200 generations to do so, whereas the most competent individuals (with a competency level of 400) did so in under 6 generations. These data demonstrate the role competency plays in non-linearly improving the rate of fitness of a population and support a clear conclusion: the higher the competency, the better the performance.

**Figure 3:**
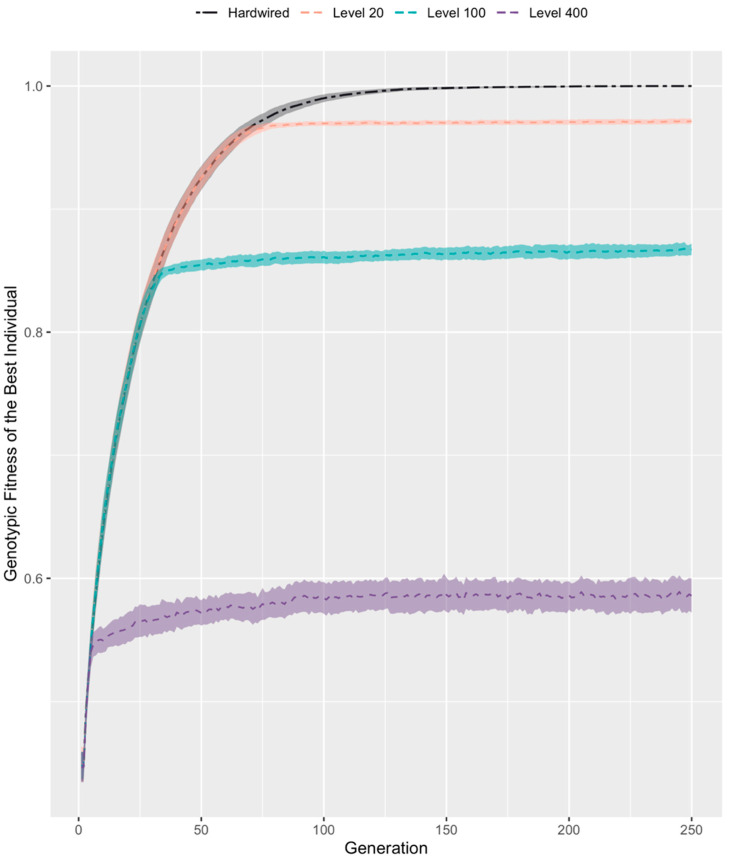
**Competency comes at the expense of reduced genotypic fitness.** Genotypic fitnesses of the best individual in three different competent populations (competency levels 20, 100, and 400) were compared with that of a hardwired population over 250 generations. Genotypic fitness is calculated by computing what the phenotypic fitness of an individual would have been if it were not allowed to enact its competencies. Shaded areas in the figure represent 95% confidence interval bands over *n* = 100 repeats.

Based on the impact of competency, one could hypothesize that progressively increasing competency would lead to a progressive decrease in selective pressure for good structural genes to appear. An embryo with high competency would have no selective pressure to improve its structural genes beyond a certain level because it can rely on its competency to re-order its cells to reach peak fitness. This is in fact what we observed (Figure 3). We compared the genotypic fitnesses of the best individual in three populations with different levels of competency (20, 100, and 400) to that of a hardwired population. In all three competent populations, genotypic fitness rose with that of the hardwired population for a few generations, after which it plateaued, indicating that at this point, the structural genes were good enough for competency to achieve a phenotypic fitness that insured selection. Further, with increased competency, the 95% confidence interval bands for genotypic fitness grew wider. Thus, as hypothesized, increasing competency in our simulation enabled excellent performance but reduced selective pressure on the embryo’s structural genes.

### 3.3. Competent Individuals Take over Mixed Populations

**Figure 4:**
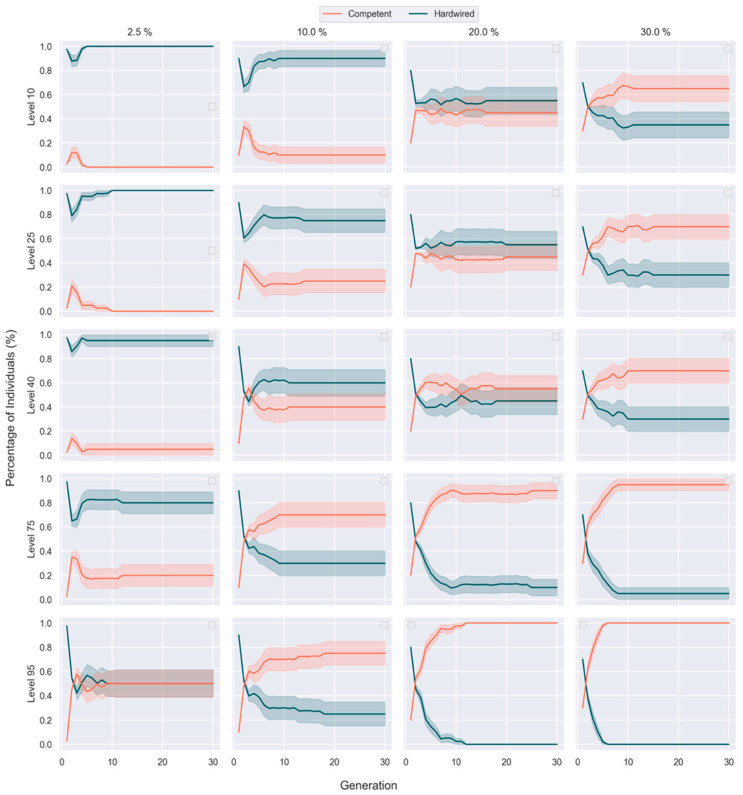
**Competent individuals dominate over hardwired individuals in a mixed setting when given adequate competency.** Each column represents the percentage of competent embryos in a hybrid population (*n* = 200 total) at initialization, increasing from left to right. Each row shows data from experiments at different competency levels, which increase from the top to bottom. Simulations were run for 30 generations. Shaded area represents variance over 20 repeat runs of each experiment.

Given these tradeoffs, we next asked how mixed populations (200 embryos per population) of competent and hardwired embryos would evolve (Figure 4). We varied both the level of competency and the percentage of competent embryos in the hybrid population at the start of the simulation. To probe the levels of competency required for embryos to dominate the population over the evolutionary simulation, competent embryos were always initialized as a minority of the starting population. Relationships between competency, initial population proportion and dominance were observed over several runs.

When competent embryos constituted just 2.5% of the initial population, they failed to dominate even at the highest level of competency tested: embryos with a competency level of 95 merely reached equal percentages with hardwired embryos. As their initial proportion in the population increased, competent embryos required progressively less competency to dominate over their hardwired competitors. At 10%, embryos with a competency level of 75 could dominate; at 20%, the competency level required for domination decreased to 40; and at 30%, competent embryos dominated with a competency as low as 10 (Figure 4). In all starting conditions that resulted in dominance of competent embryos, it occurred rapidly, in just two or at most three generations (Table 2).

### 3.4. Evolution Results in a High, Constant Level of Competency

**Figure 5:**
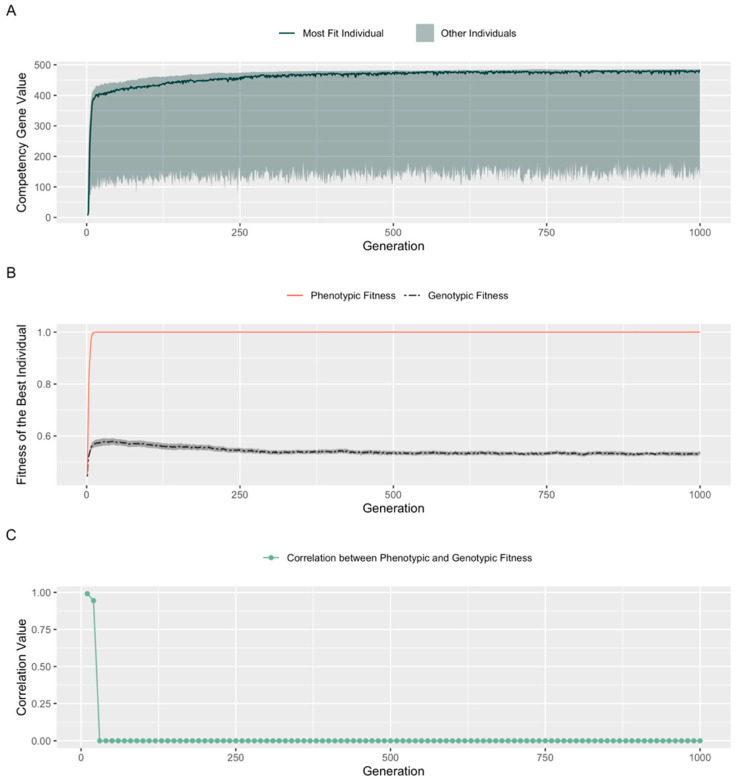
**Allowing evolution to set competency level: a perfect genome is not required to boost fitness**. Competency gene values for embryos (*n* = 100) were randomly initialized in the range of [1, 15]. Over the course of evolution each competency gene was allowed to mutate to a value in the range of [1, 500]. (**A**): Competency gene value of the most fit embryo over the course of evolution. Shaded area represents the range of competency gene values in the population. (**B**): Fitnesses of the best individual in a population of competent embryos with evolvable competency. Shaded area represents variance over 100 runs. (**C**): Correlation of the genotypic and phenotypic values of the population (shown as average values over sequences of 10 generations).

To determine how competency might spontaneously evolve over generations, we introduced competency as an evolvable trait by letting each embryo’s competency level be determined by a single ‘competency gene’ with value in the range [1, 500]. During initialization, the competency genes of all embryos were set randomly to low values in the range [1, 15]. Then, during evolution cycles, we allowed each competency gene to be mutated, potentially taking values across the range of [1, 500], and tracked the competency gene values of the best individual over 1000 generations (Figure 5). The prevalence of the competency allele rapidly rose, meandering and exploring values up to 485 during evolution (shaded area in Figure 5A) before plateauing at ~470. We provide a possible explanation for this outcome in the Discussion section.

To understand how allowing the competency gene to evolve over 1000 generations affects genotypic fitness, we looked at the phenotypic and genotypic values for the fittest individual in each generation (Figure 5B). Values for the fittest individual quickly settled at consistent configurations in which the phenotypic and genotypic fitnesses diverged considerably. This is a fascinating outcome because it suggests that a certain level of competency reduces the pressure for improvements in an embryo’s structural genes. Once selection can no longer distinguish whether fitness is achieved by a set of good structural genes or by a high competency level that compensates for a poor set of structural genes, it can only improve the population by increasing competency, not by selecting better genetics.

**Figure 6:**
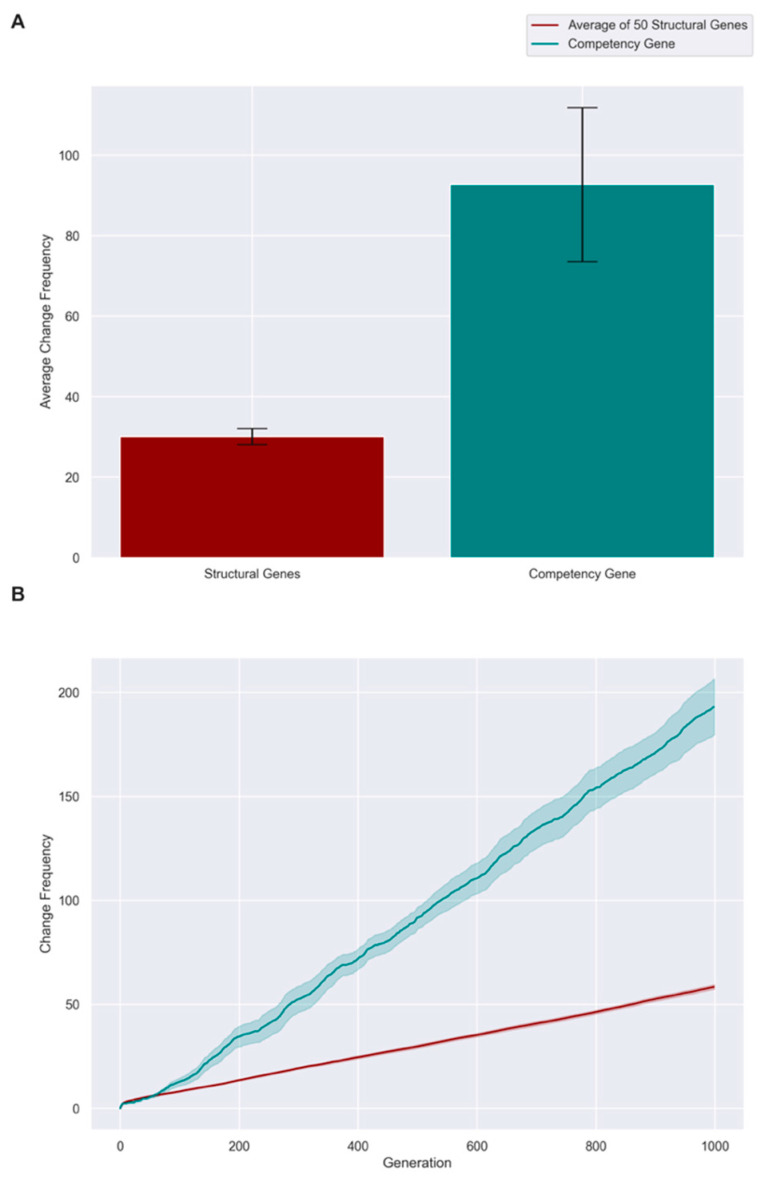
**Evolution spends a greater proportion of time tweaking the competency gene compared to any structural gene**. Employing the experimental setup of Figure 5, we checked how often changes occur within the structural genome of embryos vs. the competency gene, to determine where the evolutionary process focuses most of its effort under various conditions. (**A**): Frequency of changes that 50 structural genes undergo versus the frequency of change that 1 competency gene underwent, averaged over time. Error bars represent standard deviation over *n* = 100 repeat runs of the experiment. (**B**): Comparison of frequency of changes in 50 structural genes versus 1 competency gene, as a function of evolutionary time. The graph is cumulative, i.e., the number of changes made in the previous generation carry forward to the next. Shaded area represents variance over *n* = 100 repeat runs of the experiment.

To quantify this effect and determine how well selection, which ‘sees’ phenotypic fitness only, selects for genotypes when competency is allowed to evolve, we plotted the degree of correlation between genotypic and phenotypic fitness for all individuals in these populations (Figure 5C). Correlation dropped to 0 within about 20 generations as individuals who succeeded because of their developmental competencies rapidly dominated the population. We conclude that allowing competency to evolve disrupts the ability to select for the best structural genes. We further validated this by examining the frequency, over 1000 cycles of evolution, with which positional changes to a single ‘cell’ resulted from tweaks to the competency gene vs. from tweaks to one of the structural genes. Figure 6 shows that the frequency of changes to the competency gene was much higher than the average of all fifty structural genes across 1000 generations in our simulation.

### 3.5. A Fitness Penalty for Competency Leads to Continued Improvement of an Embryo’s Structural Genes

The Baldwin Effect [59] is the now broadly accepted phenomenon in which individual organisms can achieve greater reproductive success based on behavioral adaptations, and that these adaptations can eventually become hardwired into the genome in subsequent evolutionary cycles. Hinton and Nowlan [58] found that introducing an algorithm for behavioral learning into evolutionary simulations produced outcomes consistent with the Baldwin Effect. Our initial simulations of the evolutionary impact of cellular competency did not exhibit the Baldwin Effect. This could have been due to the fact that our minimal model did not simulate any cost associated with increasing cellular competency, and thus there was no selective pressure towards genomic changes. Although the actual energetic (or other) costs of cellular competencies are not known for any living model system, it is possible that the cellular computations required for axial patterning require additional resources over and above developmental events (competent or not) that are essential for any embryo. Thus, we next studied the effects of introducing a competency cost by penalizing the fitness of embryos in our model by a factor of their competency-value. Using penalty factors in the range of [1 × 10^−7^, 0.5], we did see a Baldwin effect: the rate of rise of genotypic fitness corresponded positively with the increase in penalty factors. For penalty factors over 0.5, the genotypic fitness rose well above the phenotypic fitness, leading to disappearance of the Baldwin effect.

**Figure 7:**
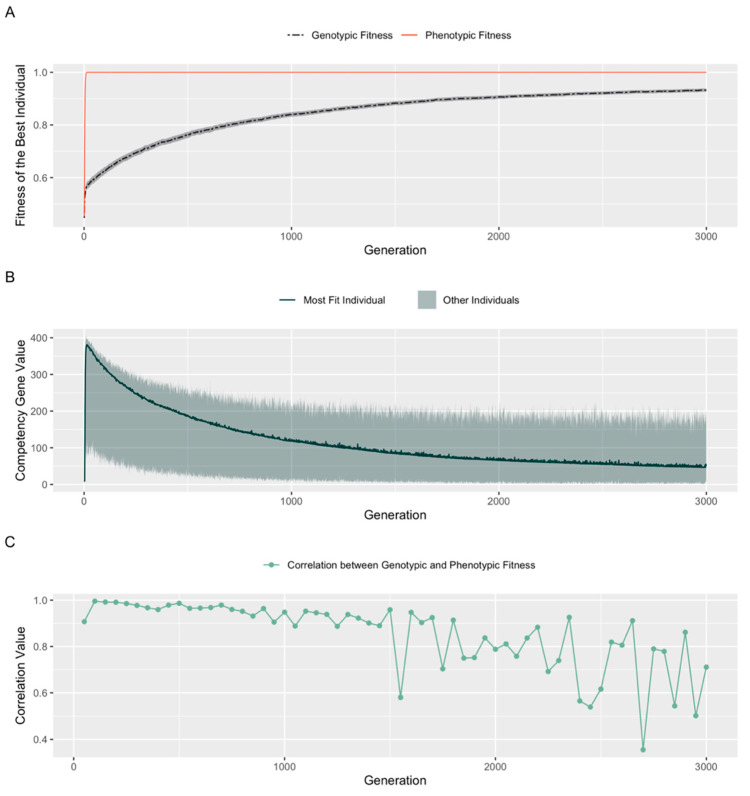
**Penalizing competency leads to its redundancy over time: the Baldwin Effect**. Competent embryos (*n* = 100) were initialized with an evolvable competency gene. At each developmental cycle, a fitness penalty of 1 × 10^−4^ times the competency value was applied. (**A**): Fitnesses of the best individual in a population of competent embryos with evolvable competency penalized by a factor of 1 × 10^−4^. Shaded area represents variance over 100 runs. (**B**): Competency gene value chosen by the most fit embryo over the course of evolution. Shaded area represents the range of competency gene values in the population at each time point. (**C**): Correlation of the genotypic and phenotypic values of the population (shown as average values over sequences of 50 generations).

The results of simulation using a penalty factor of 1 × 10^−4^ over 3000 generations are shown in Figure 7. As described above for simulations with no competency cost, phenotypic fitness reached its maximum in under 20 generations. However, unlike the previous experiment, the fitness of the structural genes did not plateau after a brief increase, but continued to improve over the course of evolution (Figure 7A). Further, as the genotypic fitness rose, selection preferred progressively lower competency values (Figure 7B). Phenotypic fitness was maintained at the maximum level, but the way in which embryos achieved phenotypic fitness evolved to value structural genes over the competency gene. Over time, selection ensured that the genotype improved to a stage where competency became redundant—the Baldwin effect [59]. We conclude that in the context of expensive competencies, selection is faced with a tradeoff between competency and the structural genome: it can either pick high competencies and bear subsequent penalties, or, it can pick low competencies and improve its structural genome. Since improving the structural genome does not bear a cost, selection prioritizes improvement of the structural genome, and over time, nullifies the effect of competency. Thus early gains based on the competency gene are later assimilated into the structural genes, paralleling what has been described previously in the context of organism-level learning [59].

## 4. Discussion

Here, we focused on a specific and novel question: the implications, for the rate and course of evolution, of a developmental process that exhibits competency at problem-solving in anatomical morphospace [59]. We produced a minimal simulation (Figure 1) that abstracted away many biological details to focus on a simple architecture: a phenotype easily optimized by traditional genetic algorithms, and a new component: competency of the individual cells to move based on interactions with neighboring cells, simulating a single body axis morphogenetic gradient of positional information [59].

### 4.1. Genotypic vs. Phenotypic Fitness: Cellular Competencies and Learning

Our approach is related to the work on the role of learning in evolution [58,60,61,62,63,64,65,66,67]. What is similar is the emphasis on genetics as the specification for a system that will then exhibit diverse behaviors that are not themselves hardcoded in the genome. Additionally, similar is the fact that DNA, as a code for specifying protein sequences, actually cannot encode directly for behavioral repertoires any more than it can directly encode morphology. What is crucially different is that traditional approaches focus on animal-scale behavior, which requires a novel and specific mechanism to evolve, such as nervous systems wired so as to facilitate specific types of learning. In our model, there is no learning needed; moreover, our system’s competency arises from a basic property of single cells: the ability to sense their neighbors, prefer those of similar type, and migrate as needed to reduce stress based on unmet expectations (e.g., intercalary regeneration in the limb [68,69,70,71]). Because cells were once themselves individual organisms and are organized into networks with homeostasis, allostasis, and homeorhesis properties [72,73,74,75,76,77], evolution is working with an agential material [24,26], which has competencies that do not need to be evolved directly (are present from the start).

However, it is likely that the cellular competency we examine here, and the behavioral learning that has been modeled by others, interact in ways that have evolutionary impact. For example, cellular activities can be the subject of behavior shaping by signals (implemented by evolutionarily-sculpted properties of the subcellular hardware such as signaling machinery and GRNs). In other words, much as organism-level learning enables an individual’s function to be molded by signals from conspecifics and parasites; similarly, cellular competencies open cells up to beneficial or detrimental signals from other cells in the organism itself that can control them via real-time triggers. It will be interesting in future work to understand how many results from the evolutionary learning field carry over to the evolutionary implications of competency, and how these two different aspects of the divergence of genotype from phenotype interact with each other in hybrid models that have both features.

### 4.2. Limitations of the Study

Our framework was more complete than many evolutionary simulations because it included an explicit developmental layer between the genotype and phenotype. It was multiscale in the sense that important changes occurred on an evolutionary scale across individuals, but also ones driven by *components* of those individuals within their lifetime—the cells, which had their own perspective and local goals. However, our system clearly omitted a huge amount of biological detail with respect to cellular mechanisms of sensing, competition, cooperation, etc. We intentionally designed a minimal model to specifically focus on a few sufficient dynamics, and this likely under-emphasized the difference between cellular competencies and, for example, effects of learning at the organism level on evolution. Fundamentally we explored a toy model virtual world in which the individual roles of selection and competency could be quantitatively dissected in the absence of confounding complexity—we sought generic laws and dynamics [78,79,80,81,82], not a simulation of the detailed trajectory of any existing biological species.

Future work will add physiological layers, diverse cell types, computation at gene-regulatory and cellular-network levels, and a multi-dimensional target morphology (e.g., 2D or 3D pattern instead of just one primary axis) to more closely model biological reality. There is also much that can be improved with respect to the specific mechanisms that cells use to implement their competency: a rich set of diverse genes will be added in the future to enable evolution to manipulate different types of local goals and competencies. Moreover, recent discoveries in transgenerational inheritance [78,79,80,81,82] suggest that barrier between the genome and the phenotype is at least somewhat porous, and the effects of propagating sort order to offspring should be investigated.

### 4.3. The Role of Cellular Competency in Evolution

We found that providing cells with a minimal homeostatic competency to improve their position in the virtual embryo results in better performance of the evolutionary search. Populations reach better fitness values faster when cellular activity is able to make up for genetic deficiencies (Figure 2). Indeed, in mixed populations, competent individuals tend to dominate and rapidly take over (Figure 4), as long as they have a minimal level of competency and/or are present in adequate numbers (Table 2). The simulation highlighted the distinction between two properties of each individual that are often conflated or obscured in simulations that do not include an explicit competency step: genotypic vs. phenotypic fitness.

Indeed, biology has many examples of evolution’s attempts to gauge genomes that it cannot see directly, for example by fluctuating asymmetry [83,84] and the near universal standards of sexual selection for left-right symmetrical features (which in turn is correlated with lack of genetic damage) [85,86,87]. In this system, we see that competency results in good phenotypic fitness but takes selective pressure off of genotypic fitness, which settles at a sub-optimal level (Figure 3).

Perhaps the most interesting aspect was the role that competency plays in exacerbating the inability of selection to evaluate the genetic material that gets passed on to subsequent generations. We observed that increases in competency made it harder and harder for selection to pick the best structural genes. Specifically, the correlation between genotypic and phenotypic fitness drops to insignificant levels very rapidly (Figure 5C). This could be expected to result in complex dynamics, because competency improves fitness of individuals but impairs the ability of the evolutionary hill-climbing search in fitness space to pick out the most elite structural genomes. Thus, we studied what happens when evolution is also allowed to control the degree of competency, which is biologically realistic since cellular capacities for sensing, computation, and action are themselves under evolutionary selection. We observed that the population drives towards picking the highest competency gene value in the population (Figure 5A), settling at a value close to 470. While this value of competency is sufficient to boost an embryo’s fitness to maximum, it is not necessary. We propose the following explanation.

Initially when evolution begins, the ordering of the cells is far enough from ascending order that a high competency gene value is required to create individuals with high fitness. At generation 20 or so, maximum fitness is achieved by choosing high competency gene values, and by simultaneously improving structural genome quality to 57% genotypic fitness. After generation 20, the genotypic fitness drops to a value of 52% and stabilizes with no further improvements. From this value of genotypic fitness, a competency value of 364 would theoretically be adequate to reach peak fitness. As a result, there is no selection pressure for evolution to always pick the highest possible competency value (i.e, a value of 480 seen in the shaded area of Figure 5A), because a value of 480 confers no additional phenotypic fitness over a value of 364. They are perceived as equal by the selection process and hence a random walk between these values would suffice. The reason we notice evolution picking values close to 480 at the end of 1000 generations could be because of a stochastic component to selection of embryos with competency gene values above 364. In our experiments, if >10% of the population have a fitness of 1.0 before selection, we pick the first 10%. This leads to the random selection of competency genes within the range of [364, 480].

In our models, we had to make a number of quantitative choices with respect to the evolutionary process. Thus, we checked how sensitive our conclusions were to these decisions via a hyperparameter scan: re-running the simulations with different choices for various hyperparameters (see Supplement S2.3.1). Specifically, we identified mutation probability and selection stringency as key hyperparameters which could influence the results of evolution. In an effort to probe their influence on the final competency gene value attained, we ran this experiment for 132 different combinations of mutation probability and selection stringency in the range of [0.2, 0.8] and recorded the stable-competency value attained for each hyperparameter combination (Figure S1 in Supplement S2.3.1). Correlation analysis revealed that a correlation of −0.4 existed between mutation probability and stable-competency-gene-value. However, no relationship was found between selection stringency and the stable-competency-gene-value. A possible reason for this could be that after generation 20, almost every embryo in the population achieves maximum phenotypic fitness, therefore there is no difference in choosing the top 20% of the population or the top 80% of the population. Mutation probability on the other hand has a direct influence on changing individual fitness, which explains its moderately significant relationship with the stable-competency-gene-value.

### 4.4. The Paradox of Robustness: Why the Animal with the Worst Genome Has the Best Anatomy

The competency of a population can be seen as granting robustness against perturbations, i.e., competency resolves aberrations in the genome and lessens the burden on evolution. The role of robustness in evolution has been a popular topic of discussion [88,89,90,91,92]. A population’s robustness is hypothesized to cause an evolutionary reduction in its adaptive performance; a sort of maladaptation caused when improved robustness traits layer on top of one another over evolutionary time and hide the underlying adaptive traits. This paradox has been shown to have broad implications on organismal design and is supposed to be a key aspect of evolution. Our results are in line with this paradox. Figure 3 is a clear depiction of the role robustness plays in hindering the quality of the genome. At each generation, increasing competency adds robustness that shields genomes which otherwise would have been culled by evolution. When compared to a population with no competency (hardwired), genomes in competent populations stabilize to a mediocre value whereas the untampered hardwired genomes rise steadily to maximum fitness. However, it must be noted that this is not necessarily a disadvantage. The paradox reveals the efficiency of competency: genomes need not be perfect; a stable threshold value of the structural genome is all that is required for competency to boost an individual’s fitness to maximum. Genetic information and problem-solving capacity of the cells work together to achieve a perfect solution to this fitness function.

This dynamic relationship between genotype and cell competency demonstrated in our simulation uniquely explains the remarkable example of planarian biology described in the introduction [19]. How can animals with a chaotic genome have such robust anatomies? We propose that planaria are an example of runaway competency: when cells get really good at making up for deficiencies in the structural genes, evolution has such a hard time selecting for the best genomes that further improvements instead increase generic competency to reach their target morphology despite perturbations. This positive feedback loop results in biological hardware that is highly successful at maintaining a specific morphology in a wide range of circumstances.

Tolerance to genetic and environmental insults is seen to some extent in other species; for example, human embryos are tolerant to being split at early stages, creating normal monozygotic twins, while mutations in important genes can sometimes be overcome by development [93,94,95]. However, in the amazingly regenerative planaria the effect was apparently much stronger. We propose this also as an explanation for another curious aspect of planaria. In every other model species, mutant lines are available—fruit flies with different number of wings or color of eyes, mice with abnormal tails, and many more genetic strains that are available from stock centers. In planaria this does not exist—no morphologically abnormal genetic strains have been reported. In fact, the only available abnormal line of planaria is a permanently two-headed form [96,97,98], which was produced not genetically but by manipulating bioelectrical signaling—the modality that is used to coordinate cellular competency [99,100,101], as is predicted by our model for species like planaria. Given their resistance to mutation, it’s unclear how speciation in planaria happens, but it should be noted that the same bioelectrical strategy that controls computation and cognition (i.e., behavioral competencies) in brains has been shown to coax genetically wild-type planaria to grow the heads appropriate to other species [102,103].

### 4.5. Genes Can Specify Direct Features, or Problem-Solving Behaviors

Of course, competency is itself carried out by molecular hardware which itself is subject to evolution and is encoded in the genome. However, it has long been clear that genotype does not uniquely determine the phenotype [104,105,106]). Development (and thus, evolution) can make use of many principles of physics (bioelectric computations, biomechanics, GRN memory, and other inherent properties [14,15,16,107,108,109,110,111,112,113,114]) that are not directly encoded anywhere but are exploited by the genome-specified machine. Our simulations study the effects of one type of such “free lunch”: cellular positional preferences and ancestral capability of motility during development, which are distinct from the environmental influences studied during typical epigenetics research. Our distinction is between structural genes (which directly specify phenotypic features) and competency genes (which specify a problem-solving machine that can exert context-sensitive activity). This is a powerful distinction for the same reason that the hardware-software distinction has driven a revolution in information technology. While the hardware (genome) is essential and important, software (competency) harnesses novel laws of physics, computation, and information processing that are not directly encoded by the hardware. This is akin to the way a logic table is implemented, but not directly encoded by, the specification of the transistors that make up a logic gate. This has been emphasized by fascinating work on the “arrival of the fittest” (evolutionary exploitation of “free lunches” provided by generic laws such as network properties [107,113,114,115,116,117,118,119]). While genes determine enzyme function fairly directly, the relationship between genes and complex morphology and behavior is extremely indirect [120]. However, the distinction between these modalities is not binary. Thus, a more nuanced future framework will quantify (and exploit) a continuum of degrees of directness with which a generative encoding determines form and function from a given informational seed.

### 4.6. Where the Hard Work Is Done: An Intelligence Ratchet

In planaria, most of the evolutionary “effort” seems to have gone into perfecting the algorithm (the ability of cells to create a normal worm morphology), vs. keeping a clean genome, because of the vicious cycle of competency increases. We found that the competency gene is changed significantly more often over the course of evolution than any structural gene (Figure 6). When gains can no longer be made efficiently by tweaking the genome (once selection cannot reliably pick out the good genotypes), all the effort goes in to increasing the competency level. This suggests the existence of a powerful ratchet mechanism in which evolution progressively becomes locked into improvements in the intelligence of the agential material with which it works, with reduced pressure on the structural genes. A positive feedback loop in which evolution increasingly puts more effort into the developmental software than perfecting the hardware points to a possible drive for scaling intelligence in morphological and other spaces [53,54,55,121,122,123]. It is possible that a drive for increased competency is an ancient and ubiquitous pressure [72], which plays out to different degrees in different biological lineages based on other aspects of their environmental and reproductive hyper-parameters.

### 4.7. The Costs of Competency

One factor resisting the runaway positive feedback for multiscale intelligence is the cost of competencies. When included in our models (Figure 7B), it induced a classic Baldwin effect of assimilation into the genome and subsequent lessening of the drive for competency. However, whether this is realistic remains to be determined by measurements in vivo that have not yet been done. On the one hand, it is reasonable to posit that specific developmental computations (that might be needed for anatomical homeostasis for example [124,125]) could carry a metabolic or other cost. On the other hand, these may be capacities that cells are already doing regardless—they may be impossible to turn off, and may represent a use of internal processes that carries no extra penalty. Examples include bioelectric signaling that controls morphogenesis via ion channels needed for housekeeping physiology and cancer suppression [99,126], and learning properties of gene regulatory networks [127,128], which are emergent and require no new mechanisms for structural plasticity. Moreover, some properties (such as behaviors and morphogenetic outcomes) may simply be too hard to encode genetically, since genes directly specify proteins—not complex anatomical states. Our simple model of a 1D positional information axis did not enable that distinction (which may have otherwise limited the Baldwin effect and kept up the pressure for competency, for the same reason that brainy and highly morphologically plastic animals have advantages, despite the possibility of assimilation).

Another reason to include a competency penalty is to account for the extra developmental time that may be needed for the cellular activities to take place. However, it is not clear that this is a fair adjustment. We know of no data to suggest that the cleverer activity of competent morphogenetic processes takes longer than is required by minimal, feed-forward developmental mechanisms. Thus, giving a hardwired individual credit for completing development faster (equivalent to the penalty for competency in our Baldwin effect experiments) may not be an accurate modeling of the biology. Thus, we believe conclusions about the Baldwin effect and the limitations on competency observed in Figure 7 should be re-investigated in future work, when the real-world costs of these processes can be measured.

## 5. Conclusions

These results suggest a diverse research program on the evolutionary interplay between biological hardware and software. We suggest that the field of basal cognition [52,53,54,57,121,122,129,130,131] is an important part of understanding evolutionary developmental biology [73,132,133,134,135], and that intelligence (problem-solving competency) was an evolutionary driver long before complex brains and muscle-driven behavior arose [52,53,56,136,137,138,139,140,141,142,143]. Beyond understanding natural evolution, we suggest that the design of autonomous robotics [144,145], synthetic life [146], and interventions for regenerative medicine [35] can all benefit from deciphering and exploiting the multiscale competency architecture so richly exhibited by living forms.

## Figures and Tables

**Table 1:** **The number of generations different populations take to break through a particular fitness threshold.** The break-through times reported are for the best individual in the population. Competency level indicates the number of swaps available to each embryo when initialized.

Competency Level	Fitness Threshold					
	0.65	0.75	0.8	0.9	0.97	1.0
No competency (Hardwired)	10	18	24	42	72	250
Level 20	9	16	21	36	55	93
Level 100	5	9	12	19	26	37
Level 400	2	2	2	2	3	5

**Table 2:** **Time taken by competent embryos to dominate over hardwired embryos when mixed together in different ratios**. Each column indicates the proportion of competent embryos in a hybrid population of size 200. The remaining embryos of the population are hardwired. Each hybrid population was evolved over 30 generations with a fixed level of competency (rows). Competent embryos are said to dominate when their prevalence rises over that of hardwired embryos and continues to rise or remains stable without dropping. Values indicate the number of generations required for competent individuals to dominate over hardwired individuals. “x” indicates no dominance.

Competency Level	Percentage of Competent Embryos			
	2.5%	10%	20%	30%
Level 10	x	x	x	3
Level 25	x	x	x	3
Level 40	x	x	3	3
Level 75	x	3	3	2
Level 95	x	3	2	2

## Data Availability

Our code is made publicly available at https://github.com/Niwhskal/CellularCompetency (accessed on 7 August 2022).

## Supplementary Materials

The following supporting information can be downloaded at: https://www.mdpi.com/article/10.3390/e25010131/s1.
