# Supplementary material for: Cellular Competency during Development Alters Evolutionary Dynamics in an Artificial Embryogeny Model
Source: Entropy (Basel). 2023 Jan 9;25(1):131. doi: 10.3390/e25010131 (PMC9858125; doi:10.3390/e25010131)
Supplement: Supplementary file 1 [file entropy-25-00131-s001.zip › entropy-1880908-supplementary.pdf]

Supplementary information for "Cellular Competency during Development Alters Evolutionary Dynamics in an Artificial Embryogeny Model"

Supplement S1

The general theme of our paper concerns the evolution of artificial embryos in silico. This section describes the nature, structure, life cycle, and the evolutionary process of artificial embryos in our simulation.

S1.1 Nature of Embryos

An artificial embryo is a single one-dimensional array of size 50. Each cell of this array carries an integer value ranging from 1 – 50 (inclusive). At initialization (i.e. generation 0), cells of this array take random values in [1-50] with repetition. We consider this one-dimensional array (i.e. the embryo) as a hypothetical biological organism with a specific "morphological structure", arising because of the arrangement of integer values in its cells. At generation 0, multiple one-dimensional arrays are initialized with random cell values, each of which possesses a different "morphological structure".

Two kinds of artificial embryos exist in our setting: "hardwired" embryos and "competent" embryos. The difference between them is in how they "develop" (described below). A "competent" embryo consists of cells capable of sensing its neighboring. It leverages its sensing capabilities to reorganize its cells in a way to boost fitness (described below). "Competency" is the capability of these embryos to carry out such reorganization. A hardwired embryo, on the other hand, does not have any such capability, nor any other capability. Their structure from birth to maturity is constant i.e., hardwired.

S1.2 Competent Embryos

A competent embryo is a one-dimensional array of size 50. The cells of this array are initialized randomly (with repetition) in the range of [1-50]. Competent embryos have a unique capability: Each cell of the one-dimensional array can sense its neighbor's (right neighbor) integer value. By doing so, each cell can swap its integer value with its neighbor. Swapping occurs in a manner so as to lead to an ascending arrangement of its cells by integer value. Since the data structure in question is an array, swaps occur by means of a bubble-sort procedure. However, unlike conventional bubble-sort which proceeds until an array is completely sorted, we carry out "restricted" bubble-sort, wherein the number of swaps is restricted by a "competency value" which is pre-specified as a hyperparameter in all experiments (Figures 2, 3, 4 in the main paper)

except in the experiment where "competency value" is made evolvable (Figures 5, 7 in the main paper.)

S1.3 Hardwired Embryos

A hardwired embryo is a one-dimensional array of size 50, the cells of which are initialized randomly (with repetition) in the range of [1-50]. Hardwired embryos do not have the capability to swap: they remain as initialized throughout a generation.

Embryos (regardless of kind) are evolved until their cells are arranged in ascending order by integer value. This specific ascending "morphological structure" is set to carry maximum fitness.

S1.4 Fitness

Based on the arrangement of integer values in a one-dimensional array (i.e the embryo), fitness of the embryo is calculated as follows:

Consider an embryo *A*, of size *n* initialized with random integer values in the range of [*1*, *n*]. Let *A(0), A(1), … , A(n)* be its elements.
We count the number of array elements which **do not** require to be swapped for the array to have ascending order. We call this the "non-inversion" count. Specifically:

$$\boldsymbol{nonInvCounts}\ (\boldsymbol{A}) = \#\{\ (\boldsymbol{A}(\boldsymbol{i}), \boldsymbol{A}(\boldsymbol{j}))\ \mid\ \boldsymbol{i} < \boldsymbol{j}\ \&\ \boldsymbol{A}(\boldsymbol{i}) \leq \boldsymbol{A}(\boldsymbol{j})\ \}$$

where $i \neq j$ and $\boldsymbol{i = 0, 1 \ldots n\text{-}1}$
$\boldsymbol{j = 1, 2, \ldots n\text{-}1}$
and where '#' = number of elements

Non-inversion counts of array A are normalized as follows:

$$\boldsymbol{normalizedCount} = \frac{\boldsymbol{nonInvCounts}\ (\boldsymbol{A})}{\boldsymbol{nC2}}$$

Finally, the fitness is reported on an exponential scale:

$$\boldsymbol{fitness} = \frac{\boldsymbol{9}^{(\boldsymbol{normalizedCount})}}{\boldsymbol{9}}$$

S1.5 Developmental Cycle: Restricted Bubble Sort

At the beginning of each evolutionary cycle, competent embryos are considered "just born"; their morphological structure having just been decided by their parents from the previous generation. Therefore, their fitness at this point of time is called the "genotypic fitness". Hardwired embryos and competent embryos both carry a genotypic fitness at the start of every evolutionary cycle.

Soon after, embryos undergo a process of development. Competent embryos carry out restricted-bubble sort to rearrange their cells in a way to boost fitness (i.e. in a way to increase ascending order of its elements). Hardwired embryos have no such capability. They end their life cycle with the same structure as that of at birth.

At the end of their respective developmental cycles, embryos become "individuals": competent embryos become competent individuals, and hardwired embryos become hardwired individuals (even if nothing changes structurally in them). At this point, the monotonicity of each embryo's array is calculated again to determine the "phenotypic fitness" of the individual. Since competent "individuals" have rearranged cells by restricted bubble sort during development cycle, their phenotypic and genotypic fitnesses diverge. In contrast, hardwired individuals do not rearrange, therefore their genotypic and phenotypic fitnesses are identical.

The process by which competent embryos swap their cells (carry our restricted bubble sort) to become competent "individuals" is as follows:

Given: **CompetentPopulation** (list of arrays), **competencyValue** (int), **SwapsCalculator** (function returning int), **BubbleSort** (function returning an array)

```
for embryo in CompetentPopulation:
    swapsRemaining = SwapsCalculator (embryo)
    defecitSwaps = swapsRemaining – competencyValue

    if defecitSwaps > 0:
        swapsToExecute = competencyValue

    else:
        swapsToExecute = swapsRemaining

    individual = BubbleSort (nTimes = swapsToExecute)

    CompetentPopulation.replace(embryo, individual)
```

S1.6 Populations for Evolution

Each experiment in our paper begins with a population of embryos. If a population contains only hardwired embryos, it is termed the "hardwired population", if it contains only competent embryos, it is termed the "competent population", and if it consists of both hardwired and competent embryos it is termed a "hybrid" or "mixed" population. For most of the simulations reported here *n*=100 embryos. The one exception is the simulations of hybrid populations, for which *n*=200.

S1. 7 Genetic Algorithm

In order to evolve populations (hardwired or competent), we iteratively pass them through three stages (Figure 1C in the main paper):

1. Selection: The fittest 10% (selection stringency) of individuals in a population are chosen to move on to the next generation. Selection occurs at the end of the developmental cycle.

2. Cross-Over: In order to repopulate a population back to its original strength we carry out a process of reproduction called cross-over. It occurs as follows: Two individuals are involved, each of these are split at a random location along their length. One half of Individual 1 is swapped with the same half of Individual 2 to give rise to two children. Figure 1C contains an illustration of this process. Note that hardwired and competent embryos do not "interbreed" in our crossover model.

3. Mutation: The repopulated population is subjected to random point mutations. We set the probability of an individual receiving a point mutation to be 0.6

   Note: It could so happen that for small sized arrays (less than 10 cells), repeated point mutations could cause the array to have identical cell values. However, given the large size of our arrays (50 in our experiments) and accounting for the cross-over of individuals, it is unlikely that random point mutations would lead to such a situation. Indeed, we did not notice any such pre-sorted array in our experiments.

Supplement S2

The following section provides details of each experiment. Each of these experiments were set up to run in the python programming language. Our code is made available at:
https://github.com/Niwhskal/CellularCompetency

S2.1
Experiment 1: Evolving a single hardwired population with three different competent populations

Common specifics of hardwired or competent population:

arraySize = 50
cell value initialization range = [1, 50]
selection stringency = 10%
number of embryos in the population = 100
max. Generation = 1000
mutation Probability = 0.6

number of repetitions = 100

Specifics of competent population (level 20):

competency value = 20 swaps

Specifics of competent population (level 100):

competency value = 100 swaps

Specifics of competent population (level 400):

competency value = 400 swaps

Phenotypic and genotypic fitnesses of the best individual in each of the four populations (one hardwired, three competent) in each generation are plotted over 250 generations (Figures 2 and 3 respectively in the paper).

Shaded areas indicate 95% confidence interval bands over 100 repeats of the experiment.

S2.1.1 Statistical significance

Based on the results of Experiment 1 (Figure 1 in the paper), it can be deduced that increasing the competency value leads to a higher rate of fitness increase compared to lower competency/zero competency (hardwired) values.

We verify this observation statistically by employing a *student's t-test*: Experiment-1 was repeated 100 times and fitness curves were compared at several generations. We compared the hardwired individual's phenotypic fitness to each of the competent individual's phenotypic fitnesses at generations 2, 10, and 20.
These specific generations were chosen because the variance in repeats around them was the greatest. At each of these generations, the conditions to carry out a *t-test* were verified: it was ensured that our repeats were gaussian distributed, and that they shared similar variances (the variance ratio between any two distributions was ensured to be less than 1:4). We used different random seeds for each experimental run to ensure that they were independent of each other.

Results from *t-tests* are as follows: (we notice the same *p*-values for generations 2, 10, and 20)

Phenotypic fitness [Competency 20] vs Hardwired	P-value << 10^{-3}
Phenotypic Fitness [Competency 100] vs Hardwired	P-value << 10^{-3}

Phenotypic Fitness [Competency 400] vs Hardwired	*P*-value << 10^{-3}

Table S1: *P*-values for comparison of different competent populations to a hardwired population at generations (2, 10, 20).

S2.2

Experiment 2: Evolving multiple hybrid populations, each composed of hardwired and competent embryos

Common specifics of a hybrid population:

arraySize = 50
cell value initialization range = [1, 50]
max. Generation = 30
selection stringency = 10%
total number of embryos (regardless of kind) = 200
mutation probability = 0.6
number of repetitions = 20

Specifics of hybrid population 1:

competency value = 10
number of hardwired embryos = 140
number of competent embryos = 60

Specifics of hybrid population 2:

competency value = 10
number of hardwired embryos = 160
number of competent embryos = 40

Specifics of hybrid population 3:

competency value = 10
number of hardwired embryos = 180
number of competent embryos = 20

Specifics of hybrid population 4:

competency value = 10
number of hardwired embryos = 195
number of competent embryos = 5

Specifics of hybrid population 5:

competency value = 25
number of hardwired embryos = 140
number of competent embryos = 60

Specifics of hybrid population 6:

competency value = 25
number of hardwired embryos = 160
number of competent embryos = 40

Specifics of hybrid population 7:

competency value = 25
number of hardwired embryos = 180
number of competent embryos = 20

Specifics of hybrid population 8:

competency value = 25
number of hardwired embryos = 195
number of competent embryos = 5

Specifics of hybrid population 9:

competency value = 40
number of hardwired embryos = 140
number of competent embryos = 60

Specifics of hybrid population 10:

competency value = 40
number of hardwired embryos = 160
number of competent embryos = 40

Specifics of hybrid population 11:

competency value = 40
number of hardwired embryos = 180

number of competent embryos = 20

Specifics of hybrid population 12:

competency value = 40
number of hardwired embryos = 195
number of competent embryos = 5

Specifics of hybrid population 13:

competency value = 75
number of hardwired embryos = 140
number of competent embryos = 60

Specifics of hybrid population 14:

competency value = 75
number of hardwired embryos = 160
number of competent embryos = 40

Specifics of hybrid population 15:

competency value = 75
number of hardwired embryos = 180
number of competent embryos = 20

Specifics of hybrid population 16:

competency value = 75
number of hardwired embryos = 195
number of competent embryos = 5

Specifics of hybrid population 17:

competency value = 95
number of hardwired embryos = 140
number of competent embryos = 60

Specifics of hybrid population 18:

competency value = 95
number of hardwired embryos = 160
number of competent embryos = 40

Specifics of hybrid population 19:

competency value = 95
number of hardwired embryos = 180
number of competent embryos = 20

Specifics of hybrid population 20:

competency value = 95
number of hardwired embryos = 195
number of competent embryos = 5

At the start of each evolutionary cycle, hardwired and competent embryos exist together. Each embryo develops into an individual according to its respective developmental cycle.

Selection occurs in a combined fashion: Phenotypic fitnesses of hardwired individuals are compared with the phenotypic fitnesses of competent individuals to determine the fittest 10% of the hybrid population.

During the process of cross-over, selected hardwired and competent embryos reproduce to repopulate the hybrid population to its original strength (200 in our experiments). Selected hardwired and competent embryos **do not** interbreed.

The repopulated population is subject to point mutations at random locations on their arrays, and the cycle repeats.

Prevalence (percentage) of hardwired and competent embryos in each hybrid population at the start of each evolutionary cycle is plot over 30 generations (Figure 4 in the paper)

Shaded areas around each curve indicates the variance over 20 experimental repeats.

S2.3
Experiment 3: Evolving a competent population with evolvable competency

Competent population specifics:

arraySize = 50
cell value initialization range = [1, 50]
selection stringency = 10%
number of embryos in the population = 100
max. Generation = 1000
mutation Probability = 0.6

number of repetitions = 100
competency = evolvable

Competency value of a competent population is set to be evolvable. i.e, each array has an extra cell at position *n+1* whose value is indicative of the competency value. At generation 0, every competent embryo (one-dimensional array) is initialized with an extra cell. This cell is randomly set to carry values in the range of [1-15]. During the first evolutionary cycle (i.e generation 1), this extra cell provides the competency value for each embryo. Its function is solely to provide this value; it is not considered part of the "morphological structure" of the array. Post generation 1, we allow this competency cell to be mutated to a value in the range of [1-500].

Fitnesses of the best individual in the population (Figure 5B), together with its respective competency value (called the competency gene value in the paper) (Figure 5A) are plotted over 1000 generations. The shaded area in Figure 5A indicates the range of competency gene values prevalent at any generation.

Since the population being evolved is competent in nature, it has two kinds of fitnesses: the genotypic fitness and the phenotypic fitness. Each of which is shown in Figure 5 (in the paper). Correlation of the genotypic and phenotypic fitnesses over sequences of 10 generations is also show. (Figure 5C)

<u>S2.3.1 Hyperparameter test</u>

Further, we assessed the role of hyperparameters on the value at which the competency gene stabilizes. Specifically, we checked the effect of mutation probability and selection stringency on the final stable competency gene value attained (Figure S1):

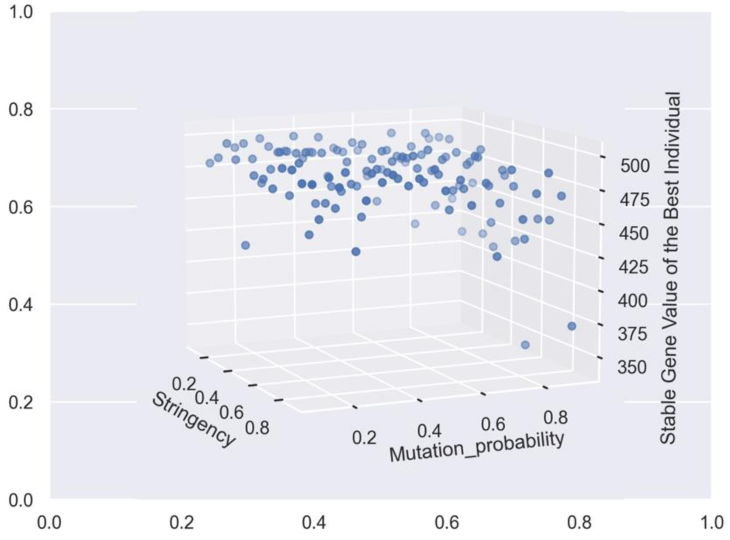

Figure S1: Results of varying mutation probability and selection stringency on the final stable competency gene value. 132 different combinations of mutation probability and selection stringency in the ranges of [0.2, 0.8] were chosen for each experimental run. Each point represents the stable

competency gene value attained for a specific combination of mutation probability and selection stringency.

Visually, we did not notice any distinct relationships between these hyperparameters and the stable-competency-gene-value attained. We carried out a correlation analysis of each of these variables to further probe the issue (Figure S2).

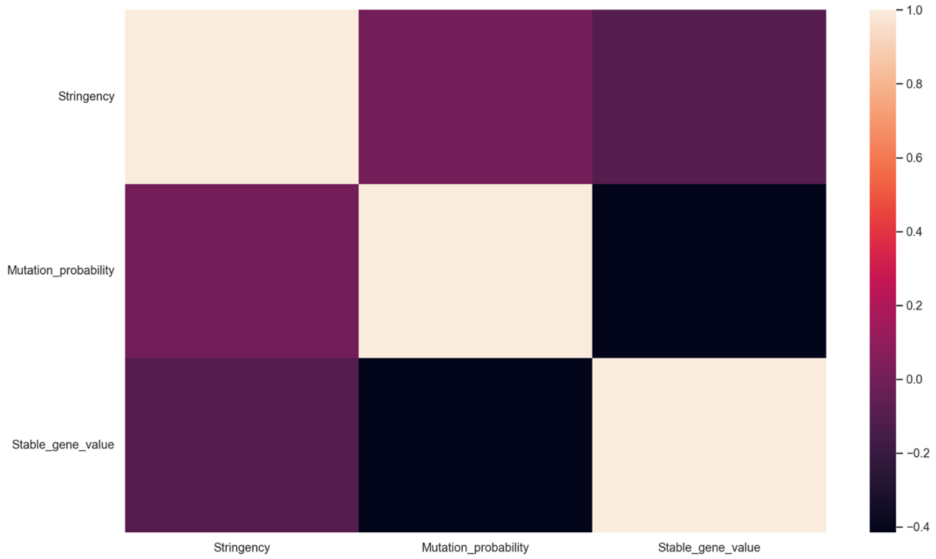

Figure S2: Correlation matrix of mutation probability, selection stringency, and stable competency-gene value attained.

The correlation matrix indicated no correlation between selection stringency and stable-competency-gene-value attained. However, a minor negative correlation (-0.4) exists between mutation probability and the stable-competency-gene-value attained.

Finally, we checked to see how often the "structural cells" of an array (the part which is assessed for its order) get modified versus how often the competency cell / gene gets modified over the course of evolution.

At each generation, each of the 50 structural cells of the array were checked to see how often they get modified. At the end of 1000 generations, we had a count of how often each of the 50 structural genes changed, and how often the competency gene of the array changed.

We took the average of these 50 structural-cell counts, and compared them to the competency-gene-change counts at each generation (Figure 6B in the paper), and at the end of 1000 generations (Figure 6A in the paper).

S2.4

Experiment 4: Evolving a competent population with evolvable competency subject to a penalty

Similar to experiment 3, a competent population with an evolvable competency gene was chosen for evolution. However, unlike the previous experiment, we penalize competency in direct proportion to the competency value.

Competent population specifics:

arraySize = 50
cell value initialization range = [1, 50]
selection stringency = 10%
number of embryos in the population = 100
max. Generation = 3000
mutation Probability = 0.6
number of repetitions = 100
competency = evolvable
competency penalty weight = 1e-04
max competency gene value allowed = 500

During the developmental cycle of a competent embryo from this population, restricted bubble-sort (specified by the embryo's competency gene) proceeds as usual. However, for every swap carried out we apply a fixed penalty. Specifically,

If the competency gene value of an embryo = $\boldsymbol{x}$

$$total\ penalty\ =\ \left(\frac{\boldsymbol{x}}{(max\_competency\_gene\_value\_allowed)}\right)\ *\ (competency\_penalty\_weight)$$

$$new\ phenotypic\ fitness\ =\ original\ phenotypic\ fitness - total\ penalty$$

This new phenotypic fitness is carried by the competent "individual" and selection is based on this modified fitness.

As before, fitnesses of the best individual, competency-gene-value of the best individual, range of competency gene values in the population, and correlation between genotypic and phenotypic fitnesses over 1000 generations are plot (Figure 7 in the paper).

In order to assess the role of the "competency penalty weight" hyperparameter, we ran several instances of experiment-4, each with a different competency penalty weight in the range of $[10^{-7}, 1]$.
Progressively increasing the penalty weight from 10^{-7} led to an increase in the rate of rise of the genotypic fitness, with the Baldwin effect being clearly evident. However, increasing the penalty weight beyond 0.5 lead to the disappearance of the Baldwin effect altogether.
